# CRISPR-induced DNA reorganization for multiplexed nucleic acid detection

**DOI:** 10.1038/s41467-023-36874-6

**Published:** 2023-03-17

**Authors:** Margot Karlikow, Evan Amalfitano, Xiaolong Yang, Jennifer Doucet, Abigail Chapman, Peivand Sadat Mousavi, Paige Homme, Polina Sutyrina, Winston Chan, Sofia Lemak, Alexander F. Yakunin, Adam G. Dolezal, Shana Kelley, Leonard J. Foster, Brock A. Harpur, Keith Pardee

**Affiliations:** 1 Department of Pharmaceutical Sciences, Leslie Dan Faculty of Pharmacy, University of Toronto, Toronto, ON M5S 3M2 Canada; 2 Department of Biochemistry & Molecular Biology, Michael Smith Laboratories, University of British Columbia, Vancouver, BC V6T 1Z4 Canada; 3 Department of Chemical Engineering and Applied Chemistry, University of Toronto, Toronto, ON M5S 3E5 Canada; 4 Centre for Environmental Biotechnology, School of Natural Sciences, Bangor University, Bangor, Gwynedd LL57 2UW UK; 5 Department of Entomology, University of Illinois at Urbana–Champaign, Urbana, IL 61801 USA; 6 Institute of Biomedical Engineering, University of Toronto, Toronto, ON M5S 3G9 Canada; 7 Department of Chemistry, Faculty of Arts and Science, University of Toronto, Toronto, ON M5S 3H4 Canada; 8 Department of Biochemistry and Molecular Genetics, Northwestern University, Chicago, IL 60611 USA; 9 Department of Chemistry, Northwestern University, Evanston, IL 60208 USA; 10 Department of Biomedical Engineering, Northwestern University, Evanston, IL 60208 USA; 11 Department of Entomology, Purdue University, 901 W State Street, West Lafayette, IN 47907 USA; 12 Department of Mechanical and Industrial Engineering, University of Toronto, Toronto, ON M5S 1A1 Canada

**Keywords:** CRISPR-Cas systems, Synthetic biology, Infectious diseases, Diagnostics

## Abstract

Nucleic acid sensing powered by the sequence recognition of CRIPSR technologies has enabled major advancement toward rapid, accurate and deployable diagnostics. While exciting, there are still many challenges facing their practical implementation, such as the widespread need for a PAM sequence in the targeted nucleic acid, labile RNA inputs, and limited multiplexing. Here we report FACT (**F**unctionalized **A**mplification **C**RISPR **T**racing), a CRISPR-based nucleic acid barcoding technology compatible with Cas12a and Cas13a, enabling diagnostic outputs based on *cis*- and *trans*-cleavage from any sequence. Furthermore, we link the activation of CRISPR-Cas12a to the expression of proteins through a Reprogrammable PAIRing system (RePAIR). We then combine FACT and RePAIR to create FACTOR (FACT on RePAIR), a CRISPR-based diagnostic, that we use to detect infectious disease in an agricultural use case: honey bee viral infection. With high specificity and accuracy, we demonstrate the potential of FACTOR to be applied to the sensing of any nucleic acid of interest.

## Abstract (web summary)

Nucleic acid sensing involving CRISPR technologies is powerful but has certain limitations, such as PAM sequence requirements and limited multiplexing. Here, authors report a CRISPR-based barcoding technology which enables multiple outputs from any target sequence, based on cis- and trans-cleavage.

## Introduction

Repurposing the components of life into the functional elements of engineered biological systems have led to great advances for the field of synthetic biology. The resulting applications have spanned domains as broad as health care^1^ (diagnostics^2^ and the development of synthetic probiotics^3^), commodities (biofuels or biomolecules^4,5^) and electronics or chemistry^6–9^. The recent development of CRISPR diagnostic strategies^10–15^ based on the non-specific *trans*-cleavage activity of these enzymes, have demonstrated specific and sensitive detection of several infectious diseases, such as the SARS-CoV-2 and Human Papilloma Virus^10,16–18^. In these applications, and for most CRISPR-Cas enzymes, a protospacer adjacent motif (PAM, found in DNA) or a PAM-like motif (called a protospacer flanking site, PFS, found in RNA), must typically be present in the amplified target nucleic acid for the pre-loaded complex CRISPR-Cas with CRISPR RNA (crRNA) to bind and induce sequence specific *cis*-cleavage activity^19,20^. This primary *cis*-cleavage event then induces the *trans*-cleavage activity used as the diagnostic output (e.g., fluorescence). Unfortunately, while PFSs are accommodating^21,22^, this dependency on PAM availability limits existing CRISPR-based diagnostics in their range of possible targets. Specifically, the vast majority of potential target nucleic acid sequences are PAM-free (94.8% in the reference human genome for the Cas9 PAM ‘NGG’^23^). Given the recent emergence of CRISPR diagnostics, most established nucleic acid-based molecular diagnostics (e.g., PCR, sequencing) are directed at sequences without consideration for PAM sites. For this reason, the development of a CRISPR nucleic acid detection technology that is independent of the PAM requirement would be a tremendous benefit to the emerging CRISPR diagnostic field by broadening the target sites possible for each disease^12^.

Further, it is increasingly important for de-centralized diagnostics to provide multiplexed detection, where a common underlying molecular machinery can simultaneously monitor multiple target sequences. This requires the generation of distinct reporter readouts for each sequence of interest, which is difficult with non-specific *trans*-cleavage. To date, adding multiplexing capacity to CRISPR-based systems has been a challenge^24,25^, requiring various CRISPR enzymes^16^, and/or separate wells^26^. Other gene circuit-based diagnostics have begun to deliver the capacity for decentralized and multiple readouts^27–29^. Taken together, if RNA-free deployment and practical multiplexing could be brought to a CRISPR-based nucleic acid sensing technology, it would enable greater capacity for disease monitoring and increase flexibility in sensor readout possibilities (e.g., colorimetric, glucometric, fluorescent).

**Fig. 1:**
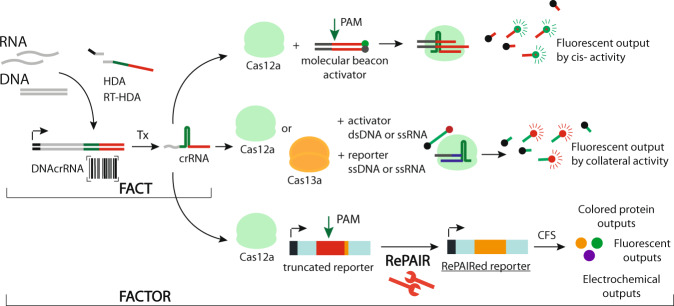
Regulation of CRISPR gene circuits through PAM-free nucleic acid barcoding amplification. The isothermal amplification of a target nucleic acid (HDA for DNA or RT-HDA for RNA) results in the generation of a barcoded, composite DNA encoding crRNA (DNAcrRNA). This functionalized CRISPR amplicon, upon transcription (Tx), produces a crRNA that can be self-cleaved and loaded by a Cas12a (or Cas13a) enzyme. The Cas12a/crRNA complex can then regulate gene circuit readouts using (*i*) sequence specific *cis*-cleavage activity (top), (*ii*) conventional diagnostic technologies based on *trans*-cleavage activity using Cas12a or Cas13a (middle), or (*iii*) CRISPR induced DNA reorganization (RePAIR system), enabling the generation of RNAs and proteins in a cell-free protein expression system (bottom).

Here we describe FACT (**F**unctionalized **A**mplification **C**RISPR **T**racing), a nucleic acid detection method based on CRISPR-functionalized isothermal amplification outputs (Fig. 1). When detected, the target nucleic acid is barcoded with a functional crRNA sequence in a single-step amplification strategy. Through downstream CRISPR *cis*-cleavage activation, the functionalized amplicon can then produce direct readouts or *trans*-cleavage induced signals. We also develop a CRISPR-based reporter system, called RePAIR (**Re**programmable **PAIR**ing), that uses CRISPR-induced DNA reorganization to activate silent gene circuits and serve as a decoding mechanism. Combining FACT and RePAIR generates FACTOR (FACT on RePAIR), which has the capacity for multiple protein-based outputs, enabling easily interchangeable fluorescent, colorimetric and electrochemical signals. FACTOR shows attomolar sensitivity to DNA targets, and the potential  for one-pot multiplexing. Moreover, FACT can be easily linked to established CRISPR diagnostic platforms through *trans*-cleavage activation. Finally, FACTOR can be rationally designed to detect viral diseases. Here, we show that FACTOR can sense agriculturally important viral infections in honey bees with an accuracy of 94.7% compared to RT-qPCR for DWV (Deformed Wing Virus) and IAPV (Israeli Acute Paralysis Virus), respectively.

## Results

### Regulation of CRISPR gene circuits through PAM-free nucleic acid amplification

**Fig. 2:**
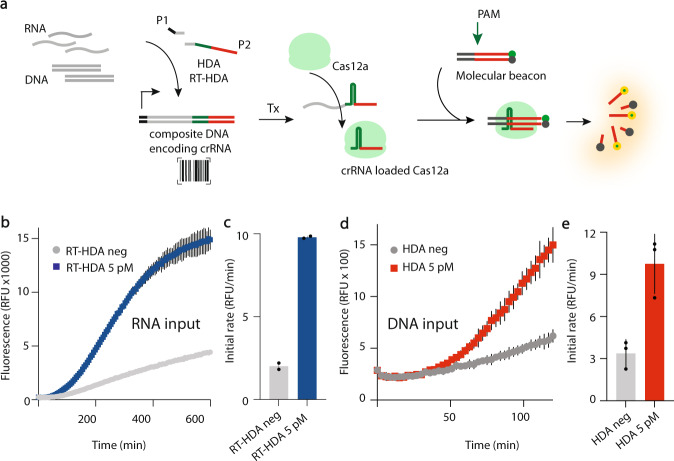
PAM-free nucleic acid barcoding amplification leads to a CRISPR induced *cis*-cleavage output. **a** Schematic of the generation of a composite DNA encoding crRNA (DNAcrRNA) upon target nucleic acid amplification using P1 and P2 primers. Following transcription (Tx), the functionalized CRISPR output product can be cleaved and loaded into Cas12a. The DNAcrRNA serves as an encoded barcode specifying which nucleic acid was amplified. The correct combination of the crRNA and molecular beacon (containing a PAM+ protospacer) generates a fluorescent output. **b** RT-HDA was performed using synthetic target RNA (Supplementary Data 1, line 93) at 5 pM or H_2_O (neg). The barcoded product was then transcribed and combined with FnCas12a and the reporter dsDNA molecular beacon. TAMRA fluorescence (543/584 nm) was monitored in real time at 37 °C in a plate reader. **c** Using the data from **b**, the initial rate of fluorescence increase within 1 h (60 min) was plotted. **d** HDA was performed using synthetic target DNA at 5 pM or H_2_O (neg). The barcoded product was then transcribed and combined with FnCas12a and the reporter dsDNA molecular beacon. TAMRA fluorescence was monitored in real time at 37 °C in a plate reader. **e** Using the data from **d**, the initial rate of fluorescence increase within 1 h (60 min) was plotted. Error bars: mean ± SD. Experiments displayed are representative of independent biological triplicates (*n* = 3). Source data are provided as a Source Data file.

CRISPR-based diagnostics typically rely on the isothermal amplification of a PAM-containing target nucleic acid^10,16^. This considerably limits sequence selection diversity for the nucleic-acid target of interest. And while CRISPR enzymes can be engineered to relax PAM recognition, broadening targeting capabilities^21,30,31^, this can be a drawback in applications where high specificity is required. To address this challenge, we set out to develop a PAM-free nucleic acid amplification strategy that could regulate a CRISPR readout system. We hypothesized that the coding sequence of a crRNA could be incorporated into a barcode on a target nucleic acid during the isothermal amplification, enabling the regulation of a CRISPR-based system. We chose to use the helicase-dependent amplification (HDA) isothermal method in combination with the well-defined Class II CRISPR enzyme *Francisella novicida* Cas12a (FnCas12a)^32,33^, capable of sequence specific *cis*-cleavage, for validation (Fig. 2a).

Using synthetic nucleic acid targets (RNA and DNA, sequences in Supplementary Data 1), we first designed the barcoding primers. The target specific forward primer (P1) encodes an overhanging T7 promoter to enable downstream transcription using T7 RNA polymerase, while the target specific reverse primer (P2) encodes for an overhanging sequence coding for a crRNA (spacer and direct repeat) (Fig. 2a and Supplementary Fig. S1). Following HDA (for DNA amplification) or RT-HDA (for RNA amplification), a composite DNA containing the PAM-free target sequence and the specific crRNA barcode is generated (DNAcrRNA). Upon downstream transcription (Tx), an RNA coding for a crRNA is produced, cleaved and loaded into FnCas12a (Fig. 2a). This nucleoprotein complex can now guide the sequence specific cleavage of reporter dsDNA, here a molecular beacon, leading to fluorescence increase.

To test our hypothesis, an RT-HDA reaction containing 5 pM of the target synthetic RNA sequence was run for 3 h to ensure amplification. The product of the amplification, incubated with FnCas12a CRISPR enzyme and a reporter dsDNA molecular beacon, was monitored in real time (Fig. 2b) and yielded fluorescent detection within 1 h (Fig. 2c). This demonstrated that the barcoding strategy was functional and could subsequently induce sequence specific *cis*-cleavage of a molecular beacon, indicating the detection of the target nucleic acid. The same experiment was also performed using a synthetic target dsDNA (Fig. 2d, e) bearing the same result. We refer to the functionalization of the amplification output with the crRNA barcode sequence, in combination with the transcription of the crRNA and the CRISPR-induced readout, as FACT (**F**unctionalized **A**mplification **C**RIPSR **T**racing).

### Reprogrammable PAIRing (RePAIR) enables a multiplexed output system

With the process of PAM-free crRNA encoded target amplification validated, we next sought to expand the capabilities of the functionalized CRISPR amplicon by linking a cell-free protein expression system (CFS) output to the sensing mechanism. CFS gene circuits have been widely used for bioproduction, rapid prototyping, and sensing applications amongst others^5,34,35^. We decided to take advantage of the multiplicity of output capabilities provided by cell-free protein expression^2,28,36^ and use FACT as a regulator of rationally designed and programmable gene circuits to generate any desired protein-based output (i.e. electrochemical, fluorescent and enzymatic).

We first developed a Homology Directed Repair system (HDR), induced by a crRNA-Cas12a complex (Supplementary Fig. S2a), building on previous work using Cas12a for the assembly of biological parts (C-Brick)^37^ and homologous recombination via SLIC (sequence and ligation-independent cloning)^38^. To do so, we engineered a non-functional coding sequence for LacZα (lipET15trcα; by deleting a segment of the coding sequence) that contained a protospacer adjacent motif (PAM) close to the deletion site (sequences see Supplementary Data 1). In the presence of a crRNA targeting the protospacer (crRNAtrcα), *cis*-cleavage should be induced and allow for a donor dsDNA fragment containing (*i*) left and right homologous arms (L-HA and R-HA) of 30 bp each to LacZα sequence and (*ii*) the missing fragment of LacZα to recombine with the truncated LacZα (Supplementary Fig. S2a). The product of the recombination, now containing a functional LacZα sequence, could then be tested in a paper-based colorimetric CFS reaction^34^ for activity. Upon complementation of LacZα and LacZΩ (supplemented into the CFS) the resulting active LacZ complex should cleave its substrate, chlorophenol red-β-D-galactopyranoside (CPRG). The resulting color change from yellow to purple can be monitored using absorbance at 570 nm.

As shown in Supplementary Fig. S2b, the proposed method successfully generated a colorimetric LacZ signal, demonstrating effective homologous recombination induced by Cas12a. Moreover, the background signal and observed leakiness of the system was not significant (ns, Supplementary Fig. S2b). We then optimized the system by varying the length of the HA and found that HA lengths of 40 bp significantly increased levels of in vitro HDR (Supplementary Fig. S2c). Although we could successfully recapitulate the LacZ enzymatic signal, this strategy ultimately failed when we extended the demonstration from a RNA molecule inducing recombination (crRNAtrcα) to a DNA molecule (DNAcrRNAtrcα). While the reasons for this are unclear, the general demonstration was a successful proof of concept for readout generation and led us to rethink our recombination system. We then designed the Reprogrammable PAIRing system (RePAIR), which allows us to use both RNA (crRNA) and DNA (DNAcrRNA) molecules as inputs.

**Fig. 3:**
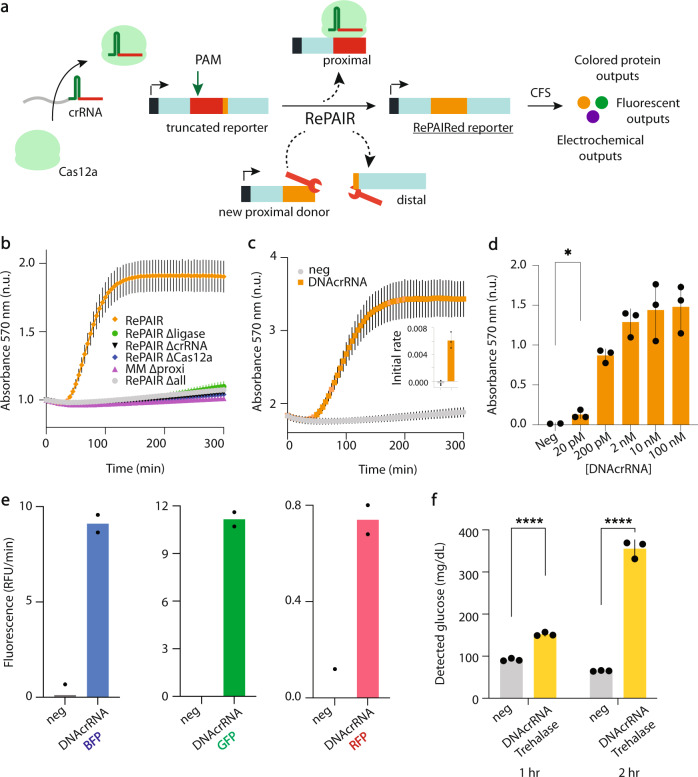
Reprogrammable PAIRing (RePAIR) enables a multiplexed output system. **a** Schematic of RePAIR, a CRISPR induced DNA reorganization process for multiplexed outputs in a cell-free protein expression system (CFS). **b** Validation of RePAIR, using a crRNA. Here, reactions contained a truncated LacZα coding sequence and were supplemented with all the components needed for RePAIR (orange) or, alternatively, selected components were removed (Δcomponent). The product of RePAIR was added to a CFS reaction. Absorbance was monitored at 570 nm in a plate reader to reflect functional RePAIRed LacZα. **c** Validation of RePAIR using a synthetic DNAcrRNA. Truncated LacZα and all the components needed for RePAIR were added to the reactions, with (orange) or without (neg-H_2_O, gray) the addition of the DNAcrRNA. The product of RePAIR was added to a CFS reaction. Absorbance was monitored at 570 nm in a plate reader to reflect functional RePAIRed LacZα. Insert shows the initial rate of absorbance within 1 h. **d** Various concentrations of DNAcrRNAtrc3a, or H_2_O (neg) were added to the RePAIR reaction to test sensitivity. Products of RePAIR were added to a CFS and absorbance was monitored at 570 nm in a plate reader. *p = 0.0188. **e** RePAIR reactions containing truncated blue fluorescent protein (BFP, lipET15trcBFP), truncated green fluorescent protein (GFP, lipBRtrcGFP), or truncated red fluorescent protein (RFP, lipET15trcRFP) were supplemented with H_2_O (neg) or with their respective DNAcrRNA. Products of RePAIR were added to a CFS reaction. Fluorescence was monitored at 399/456 nm for BFP, 485/530 nm for GFP and 584/607 nm for RFP. **f** RePAIR reactions containing truncated trehalase (lipET15trcTre) coding sequence were supplemented with H_2_O (neg) or with the matching DNAcrRNA. Products of RePAIR were added to a CFS. Production of glucose was monitored at 1 h and 2 h on a glucose meter. ****p < 0.0001. Error bars: mean ± SD. Experiments displayed are representative of independent biological triplicates (*n* = 3). Statistical test used for data analysis was a two-tailed unpaired t tests. Source data are provided as a Source Data file.

With RePAIR-based DNA reorganization, the reporter is engineered to be non-functional by removing a short segment of the coding sequence downstream of a FnCas12a PAM sequence (TTA). The deletion generates a synthetic protospacer (24 bp downstream of the PAM) that is not found in the non-engineered LacZα reporter gene (Fig. 3a, red). By programming the spacer of the crRNA to match the synthetic protospacer, sequence-specific *cis*-cleavage of the non-functional reporter should be induced in presence of FnCas12a. Furthermore, we take advantage of two additional features of the Cas12a enzyme. First, the ability of Cas12a to stay bound to the cleaved proximal side of the target dsDNA while releasing the distal side^39^. Second, the staggered cut generated by Cas12a^32^ distal to the 5’ PAM sequence on the targeted non-functional dsDNA reporter. We hypothesized that if the RePAIR reaction was supplemented with a new proximal donor containing the deleted base pairs, and a ligase to enable ligation of staggered cuts, a repaired functional reporter gene could be assembled.

We first tested the RePAIR system using a LacZα reporter as the output. Using a crRNA as the input of the gene circuit, and in the presence of all reaction components (truncated LacZα: lipET15trc3α, ligase, proximal donor strand LacZα, crRNAtrc3α and Cas12a), we detected an increase in absorbance over time (sequences of nucleic acid components can be found in Supplementary Data 1). Sequential removal of key components of RePAIR and side-by-side comparison of reactions demonstrated the successful DNA reorganization of the LacZα reporter from a non-functional coding sequence to a functional state, including its concomitant expression in CFS well within 1 h (Fig. 3b and Supplementary Fig. S3a). Building on these results, and to link the RePAIR system to the output of the barcoding amplification (FACT), we then demonstrated that RePAIR could be induced using a synthetic DNAcrRNA input (using DNAcrRNAtrc3α, Fig. 3c) with a signal clearly detected within the first hour (Fig. 3c, insert). We also tested the sensitivity of RePAIR to crRNA (using crRNAtrc3α, Supplementary Fig. S3b, sensitivity to 600 pM) and DNAcrRNA inputs (with DNAcrRNAtrc3α, Fig. 3d, sensitivity to 20 pM) in the absence of isothermal amplification, demonstrating that sensitivity of this recombination system equals that of other CRISPR nucleic acid sensing platforms^12^.

Wanting to evaluate the speed of RePAIR, using the same crRNA or DNAcrRNA inputs at 100 nM, we incubated RePAIR reactions for 5-, 15-, 30- min or 1 h at 37 °C (component sequences in Supplementary Data 1). An aliquot from each timepoint was then added to CFS to determine maximum initial rates within the first hour, with results showing all the RePAIR reaction timepoints yielded productive LacZα reorganization (Supplementary Fig. S4a). Monitoring CFS reactions for 120 min, all RePAIR reactions induced by crRNA (blue) or DNAcrRNA (red) yielded comparable absorbance reads (Supplementary Fig. S4b), allowing us, if needed, to significantly shorten the RePAIR reaction without any detection loss. It is also worth noting that at equal input concentrations, the DNAcrRNA performed better than the crRNA in the RePAIR reaction, possibly due to the amplification of the signal through the transcription of additional crRNA from the T7 promoter on the dsDNA. We refer to this combination of FACT and RePAIR DNA reorganization as FACTOR (FACT on RePAIR).

To broaden output capabilities, we designed four supplementary constructs, three fluorescent proteins and one enzyme enabling electrochemical detection. For all fluorescent proteins, we successfully detected the repaired reporter within 1 h of starting the CFS reaction (Fig. 3e). For the electrochemical output, and building upon our recent demonstration of the glucose meter as an interface for point-of-care gene circuit diagnostics^28^, we designed a RePAIR system for the *tre37A* trehalase gene. This enzyme converts a trehalose molecule into two molecules of glucose. Upon correct DNA reorganization of the *tre37A* reporter gene, glucose was generated and detected on the glucose meter (Fig. 3f). Taken together, these results show how RePAIR-mediated DNA reorganization can be adapted to virtually any sequence, demonstrated here using three different outputs mechanisms (colorimetric, fluorescent and electrochemical) with reporter proteins of LacZα, BFP, GFP, RFP and trehalase (Fig. 3).

#### One-pot multiplexing capabilities

**Fig. 4:**
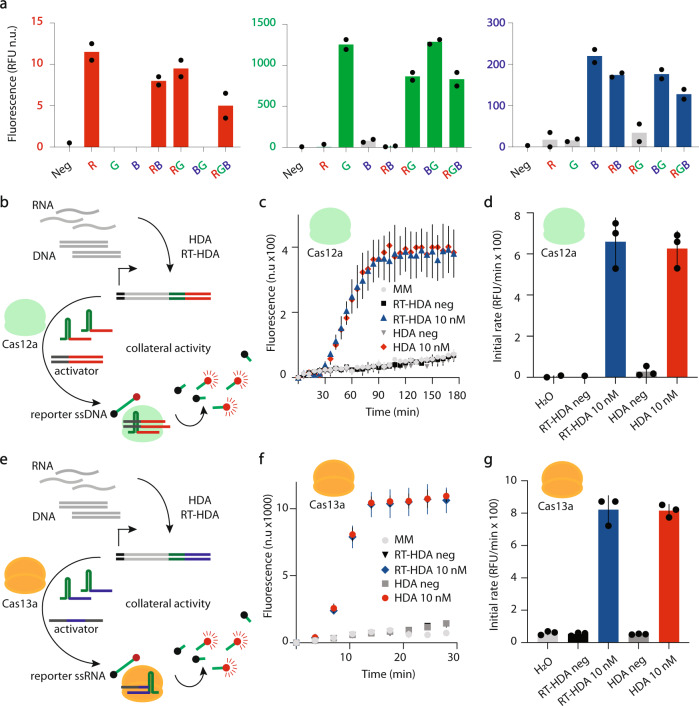
One-pot multiplexing of RePAIR and compatibility with *trans*-cleavage-based CRISPR outputs. **a** A one-pot RePAIR reaction was set up containing all truncated coding sequences, proximal donor strands and enzymes. DNAcrRNA(s) inducing RePAIR were added alone or in combination (R or G or B or RB or RG or BG or RGB, each at 100 nM). Products of RePAIR reactions were added to a CFS in technical duplicates to a 384-well plate. Each well was monitored in the three fluorescent channels (red, green, blue) to record signal increase induced by RePAIR for all the combinations. Results were plotted at the 2 h timepoint of CFS. **b** Schematic of nucleic acid sensing using the functionalized amplification (FACT) product with Cas12a enzyme in *trans*-cleavage activity. The activator is a dsDNA and the reporter is a ssDNA linked to a quenched fluorophore (all sequences in Supplementary Data 1). **c** Products of the isothermal amplification were directly added into the *trans*-cleavage readout reaction and fluorescence was monitored in real time at 536/576 nm. **d** Using data in **c**, initial rates were plotted within the first 45 min. **e** Schematic of nucleic acid sensing using the functionalized amplification (FACT) product with Cas13a enzyme in *trans*-cleavage activity. The activator is a ssRNA and reporter is a ssRNA linked to a quenched fluorophore. **f** Products of the isothermal amplification were directly added into the *trans*-cleavage readout reaction and fluorescence was monitored in real time at 490/520 nm. **g** Using data in **f**, initial rate was plotted within the first 14 min. Error bars: mean ± SD. Experiments displayed are representatives of independent biological triplicates (*n* = 3). Source data are provided as a Source Data file.

We next tested whether RePAIR is compatible with one-pot, multiplexed DNA reorganization for parallel output detection. A single mix reaction was set up containing the three non-functional fluorescent reporters (truncated RFP, BFP and GFP at equimolar DNA 100 nM concentrations), along with all of the respective donor proximal strands and enzymes (Cas12a and ligase; component sequences in Supplementary Data 1). Synthetic DNAcrRNAs were added to the mix to induce sequence-specific RePAIR, individually or in combination, incubated for 1 h, and then added to the CFS to enable protein expression. After a 2-h incubation, specific fluorescence signals were detected in each of the corresponding channels where the related DNAcrRNAs were added, demonstrating high specificity and capacity for one-pot RePAIR-based multiplexing (Fig. 4a).

#### Functionalized CRISPR amplification products as global connector to alternative CRISPR diagnostic platforms

Having established that functionalized barcode amplification products can be used directly for *cis*-cleavage outputs (Fig. 2) and induce RePAIR as an output for nucleic acid sensing (Fig. 3), we next examined whether barcoded amplicons could also be linked to *trans*-cleavage-based CRISPR diagnostics^10,11^. Using synthetic target RNA or DNA encoding for DWV, we began by performing isothermal barcoding amplification. The CRISPR functionalized products were then added to collateral cleavage fluorescent output reactions. In this context, we tested both Cas12a (Fig. 4b–d) and Cas13a (Fig. 4e–g; component sequences in Supplementary Data 1). Using Cas12a, the reaction contained an activator dsDNA as well as the quenched fluorophore ssDNA (Fig. 4b). We observed a rapid fluorescence increase in the Cas12a reactions containing either target DNA or RNA, compared to their negative controls well before 1 h (Fig. 4c and initial rate within 45 min Fig. 4d). This confirmed that, as with previous CRISPR-based sensors, collateral cleavage provides a suitable readout to FACT barcoded nucleic acids. We then moved to Cas13a, another widely utilized enzyme in CRISPR diagnostic platforms. Primer overhangs containing the direct repeat and spacer length characteristic of this enzyme were designed. Here again, a functionalized CRISPR amplicon was used in a collateral cleavage reaction containing Cas13a enzyme and its substrate, a reporter ssRNA containing a quenched fluorophore. In the presence of RNA or DNA synthetic target sequences encoding for DWV, a rapid fluorescence increase was detected in less than 10 minutes (Fig. 4f, and maximal initial rate within 14 min Fig. 4g).

### Diagnostic application of FACTOR

**Fig. 5:**
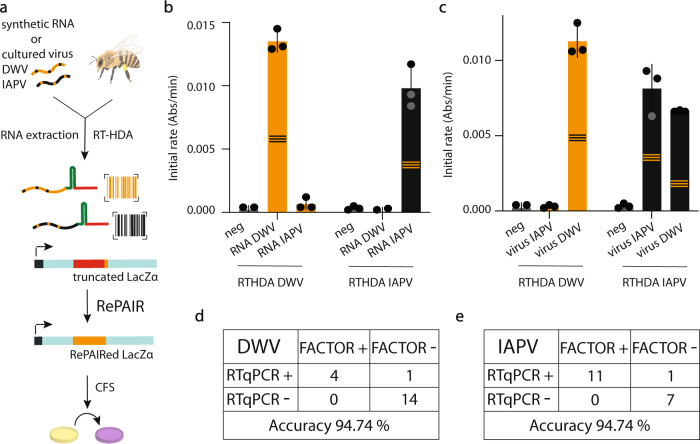
Diagnostic application of FACTOR. **a** Schematic of the FACTOR workflow using synthetic RNA, cultured viruses or whole bees for detection of DWV and IAPV nucleic acids. Following RNA extraction, isothermal RT-HDA amplification was performed, followed by RePAIR using LacZα as reporter. RePAIR products were added into CFS in technical triplicates and absorbance at 570 nm was monitored over time. **b** Using synthetic in vitro transcribed RNA, isothermal amplification was performed in solutions containing H_2_O (neg) or DWV synthetic RNA or IAPV synthetic RNA. Following RePAIR, the initial rate of absorbance within 1 h of CFS is plotted. Biological triplicate experiments were performed in technical triplicate (*n* = 3), with **b** showing one representative experiment. Error bars: mean ± SD. **c** Using cultured viruses, isothermal amplification specific for DWV or IAPV was performed on solutions containing H_2_O (neg) or extracted RNA from both cultured viruses. Following RePAIR, initial rate of the absorbance within 1 h of CFS is plotted. Error bars: mean ± SD of technical triplicate. RT-qPCR were performed in parallel (Supplementary Fig. S6). RNA extracted from whole bee lysates were used for FACTOR-based diagnosis of DWV (**d**) or IAPV (**e**) infections with parallel RT-qPCR to determine the accuracy. The honey bee design in Fig. 5 was created using a BioRender.com. Source data are provided as a Source Data file.

Finally, we sought to evaluate the potential of FACTOR for infectious disease detection in an agricultural use case. We applied our nucleic acid sensing technology to honey bee virus detection. Honey bees are critical pollinators worldwide, and, with an estimated 30% of agricultural crops depending on them, their economic and environmental impact is tremendous^40^. Unfortunately, honey bees have been heavily affected by pathogenic infections, such as viruses, that impact their productivity, ability to sense direction, life span and can cause sudden collapse of entire colonies^41,42^. Further, these viruses can ‘spillover’ from honey bees to infect native bee populations^43^. Currently, definitive diagnosis of viral infection in bees is done using laboratory-based RT-qPCR^44^, making surveillance impractical and expensive for bee managers, such as beekeepers. To demonstrate the potential of FACTOR in agricultural diagnostics, we focused on two critical honey bee viruses: Deformed Wing Virus (DWV) and Israeli Acute Paralysis Virus (IAPV) (Fig. 5a).

Using serial dilutions of synthetic RNA or DNA encoding for DWV (amplified from synthetic DNA), we began by testing the sensitivity of FACTOR. After a 90 min isothermal amplification (RT-HDA or HDA), followed by a 1 h RePAIR reaction with Cas12a, we were able to detect the synthetic target sequences at 500 fM and 50 aM, respectively (Supplementary Fig. S5). Using synthetic RNA sequences from DWV and IAPV (10 nM RNA, transcribed from synthetic DNA), we then tested the specificity of FACTOR (Fig. 5b). Using the maximum initial rate of LacZ signal within the first hour, the reaction provided clear selective detection of IAPV and DWV RNAs.

Finally, to provide a proof-of-concept for the viral detection, we used RNA extracted from viruses amplified in honey bee pupae (Fig. 5c) and from infected whole bees (Fig. 5d, e), and compared the performance of FACTOR to RT-qPCR (the current diagnostic gold-standard). Isolating pure honey bee virus (only DWV or only IPAV) is challenging and samples are often cross-contaminated with multiple viruses^45,46^. As shown in Fig. 5c, and as confirmed by RT-qPCR (Supplementary Fig. S6a), IAPV cultured virus contained only IAPV RNA, however DWV cultured virus contained both DWV and IAPV RNAs (in accordance with DWV virus preparation containing >95% DWV and <5% IAPV). Additionally, wild-caught honey bees are often infected with DWV and therefore inoculation with IAPV can lead to co-infections. Finally, 19 bees were individually analyzed for DWV and IAPV infections using the two methods. Compared to RT-qPCR (Supplementary Fig. S6b, c and Fig. 5d, e) and using initial rates within one hour (as per Fig. 5b, c), FACTOR+ was defined as greater than three SD above control signal. FACTOR successfully diagnosed 18 of the 19 bees in the DWV test, with one false negative (Fig. 5d; Supplementary Fig. S6b, c, Bee 13, red). Similarly, FACTOR successfully diagnosed 18 of the 19 bees in the IAPV test, with one false negative FACTOR for IAPV (Fig. 5e; Supplementary Fig. S6b, c, Bee 2, red). In the case of Bee 5, which had a high Ct value of 34, initial rates were analyzed during 2 h for FACTOR to be in agreement with RT-qPCR (Supplementary Fig. S6b, c, bottom graph). FACTOR results demonstrated an accuracy of DWV and IAPV diagnostic of 94.7 %.

## Discussion

Here, we report the development of FACT, a CRISPR-based nucleic acid tracing system, relying on an engineered CRISPR-functionalized isothermal amplicon. Importantly, this system is independent of any PAM sequence requirements in target nucleic acids. The output does not serve as the substrate of CRISPR, but rather acts as a regulatory element for programmable gene circuit applications. This addresses a key limitation of conventional CRISPR diagnostics^22^, where the presence of a PAM in the target nucleic acid is a prerequisite and often limits the number of detection sites. By barcoding the nucleic acid of interest with the sequence of a crRNA, we can now choose any sequence within diagnostic targets, which we anticipate will enable further expansion of the field of CRISPR-based diagnostics. The flexibility of the system also allows users to choose the CRISPR enzyme that best suits their needs (Cas12a or Cas13a) while maintaining their target site of interest, simply by reprogramming the crRNA sequence with the appropriate characteristics. We further demonstrated that FACT can guide and regulate the *cis*- and *trans*- cleavage activity of established CRISPR diagnostic platforms (Cas12a/Cas13a)^10,11^, providing a global linker between systems^10–12^. Moreover, FACT and the subsequent induced cleavage do not rely on the presence of crRNAs in the solution (free or in complex with a CRISPR enzyme). This is an important consideration for the stability of CRISPR-based diagnostics during storage and deployment to point-of-care applications.

RePAIR similarly introduces alternate capabilities to in vitro CRISPR-based circuits with a *cis*-cleavage based mechanism enabling sequence-specific DNA reorganization of theoretically any protein-based output. In conjunction with FACT, we see RePAIR as a global converter to link CRISPR activity to a broad range of gene circuit-based outputs for portable diagnostics. As we demonstrate, the direct output of amplification can be used to control in vitro transcription and translation, catalyzing fluorescent, colorimetric and electrochemical signals (GFP, RFP, BFP, LacZ and trehalase, Fig. 3b–f).

Demonstrating the simplicity of the system, we also show that RePAIR can enable one-pot, multiplexed gene reorganization events using a single CRISPR enzyme (Fig. 4a). Further, by combining the systems to create FACTOR, a simple shift of the crRNA sequence can enable a shift from fluorescence or colorimetric outputs to formats compatible with portable systems like the glucometer^28^. This has potential in the design-test process where gene circuit prototyping can be done using a simple fluorescent assay and then outputs changed for practical deployment. In the application of FACTOR to honey bee diagnostics, we found that the nucleic acid tracing technology can provide specificity and accuracy comparable to RT-qPCR, suggesting it may be useful for in-field diagnostics and the detection of other nucleic acid-based targets.

While we demonstrate a series of increased capabilities, and strong performance of the technology, these applications for CRISPR-mediated regulation are still at the proof-of-concept stage. For example, although the combined approach significantly increases the output diversity for CRISPR-based diagnostics and resolves the need for a PAM site in the specific target sequence, it also has the potential to limit the single-nucleotide resolution available with other CRISPR diagnostic tools. This is a result of directing the CRISPR activity to a synthetic sequence rather than solely to a copy of the pathogen’s genome. However, as with PCR screening, with some optimization, it is possible that single-nucleotide resolution may be restored.

To enable point-of-care deployment, we see opportunities in optimizing the speed of some steps, like target amplification or the CFS, perhaps through buffer optimization, as well as a potential to reduce the number of steps needed to perform the test. Moreover, with the cost of synthetic DNA drastically dropping, the low cost of producing in-house enzymes and the isothermal format, we see the potential for FACTOR to provide important advantages over conventional diagnostic methods. Beyond in vitro diagnostic applications, and looking toward the future, we are also curious about the potential of linking these concepts to in vivo gene expression by integrating a spacer and direct repeats into the UTR of mRNAs of interest for Cas-protein-mediated expression of reporter or functional proteins via RePAIR.

We also envision the use of DNAcrRNA as a molecular barcode, where FACT and/or RePAIR could facilitate the use of DNA to label objects. In such applications, DNA labels could be used to authenticate high-value commodities like art, food, textiles, oil, or drugs^47,48^ (Fig. S7). The concept is analogous to optical barcoding systems, such UPC or QR codes, but with the advantage that a DNA barcode is more challenging to replicate and can be embedded in or on any product, making tampering or fraud more difficult. Moreover, as a DNA molecule, such barcodes would be highly stable, and contain high combinatorial capabilities. It would also be interesting to merge the optical and DNA labeling concepts, with the development of a molecular QR code as an accessible and multiplexed readout system.

On a technical level, FACT and RePAIR expand the field of CRISPR-based diagnostics with the concepts of target barcoding and decoding, respectively. While FACT links sequence recognition to established CRISPR reporter methods, such as collateral cleavage induced fluorescence, FACTOR introduces the ability to control multiplexed protein expression in response to upstream sequences. There are many contexts where such control of in vitro protein synthesis could be exploited, including the possibility where diagnostic outcomes could direct the production of a protein-based therapeutic treatment. More broadly, the ability of FACT to serve as a global connector, converting sequence inputs into molecular signals (cleavage, RNA, proteins) provides the community with an enabling lever for engineering biology. We are keen to see how this work will integrate into the ever-growing toolbox of CRISPR technologies and platforms promoting low-cost and decentralized health care.

## Methods

### Ethical statement

All work with honey bee virus infections at the University of Illinois is done per protocols approved by University of Illinois Division of Research Safety, Institutional Biosafety Committee project IBC-198.1.

### Plasmids and oligonucleotides

#### Oligonucleotides

All oligonucleotides were ordered through Eurofin or Integrated DNA Technologies (IDT), as Gblocks, ultramers or primers. Molecular beacons were ordered as ssDNA with the fluorophore (IDT, 6-FAM) or quencher (IDT, IowaBlack FQ) attached respectively to the 3’ or the 5’ end. Bottom strands of the RePAIR donor proximal strand were ordered with 5’-phosphate to enable ligation during RePAIR.

Synthetic DNA for DWV, IAPV and rpl8 sequences were ordered and amplified by PCR (NEB Q5 M0491L) using their corresponding primers (Supplementary Data 1).

For in vitro transcription, synthetic DNA were ordered with a T7 promoter appended to their sequences or the T7 promoter was added by the forward primer used during PCR (Supplementary Data 1).

#### Plasmids

pBR939b (NovoPro, V009103) containing GFP coding sequence was a gift from the Collins lab. LacZα and LacZΩ were gifts from the Green lab. LacZα coding sequence was inserted into a pET15b backbone (Invitrogen). RFP (http://parts.igem.org/Part:BBa_E1010) (group USAFA iGEM 2019) and BFP (http://parts.igem.org/Part:BBa_K592100)^49^ coding sequences were parts from iGEM and inserted into pET15b (Invitrogen) backbone. pY002 was a gift from Feng Zhang (Addgene plasmid #69975)^32^ and the coding sequence for FnCas12a was amplified by PCR and inserted in a pET15 backbone. Trehalase was purchased from DNASU (BGH37_B01a).

### Chemicals

Unless otherwise noted, all chemicals were purchased from Sigma-Aldrich (St. Louis, MO, USA) or BioShop Canada Inc. (Burlington, ON, Canada).

### RNA synthesis and extraction

Target synthetic RNAs and crRNAs were generated using the T7 HighScribe InVitro transcription kit (NEB, E2040S), following the manufacturer’s instruction, overnight at 37 °C. RNA from bees or cultured viruses were extracted using TRIzol reagent (Thermofisher #15596026) following manufacturer’s instructions and resuspended in water.

### (RT)-HDA amplification

Reactions were assembled following manufacturer’s protocol, with minor modifications. Briefly, 9 µL reactions were assembled with 3 mM of MgSO_4_, 40 mM NaCl, 2% RNAse inhibitors (NEB, M0314L), 100 nM of each primer (see Supplementary Data 1). If RT-HDA was run, 0.1 µL (20 U) of Superscript IV RT enzyme (Invitrogen, #18090010) was supplemented. 1 µL of samples containing synthetic RNA/DNA, or 500 ng of extracted RNA from cultured viruses or from crushed bees was then added for a final volume of 10 µL, with 10 µL of oil layered on top of the reaction. Reactions were incubated between 30 min and 3 h at 65 °C. Specified concentrations represent the various concentrations of the solutions before addition to the amplification reaction.

If the output of the amplification was to be used for RePAIR or *cis*-cleavage applications, following amplification, 2 µL of the product was treated with 0.6 µL of Proteinase K (Thermofisher, EO0491) for 10 min at 37 °C and heat inactivated for 5 min at 90 °C. Heat inactivated products were used for downstream experiments at 5% v/v.

If the output of the amplification was to be used for *trans*-cleavage application, the product of the amplification was directly used in the downstream experiment at 5% v/v.

### CRISPR Cas12a *cis*-cleavage

*Cis*-cleavage experiments were performed using the proteinase K treated outputs of isothermal amplification. Briefly, reactions of 9 µL were assembled in KGB buffer^33^ (100 mM potassium glutamate, 25 mM Tris-acetate (pH 7.5), 500 µM 2-mercaptoethanl, 10 µg/mL BSA), containing: 5% v/v of the output amplification, 2 µM FnCas12a, 1 µM dsDNA reporter molecular beacon, 2 mM NTPs (NEB, N0450S), 1 U/µL of T7 RNA polymerase (Thermofischer, EP0111), 2 U/µL of RNAse inhibitors (NEB, M0314L).

Technical duplicates of 4 µL for each reaction were run in a 384-well plate (Corning #3544), monitoring TAMRA fluorescence (543/584 nm) on a plate reader (BioTek, Gen5 3.02. Fisher Scientific BTNEO2M).

### Homologous direct recombination-based RePAIR

Truncated linear pET15a (lipET15trca) (20 nM) was incubated with FnCas12a (2 µM), MgCl2 (10 mM), repair fragment containing the homologous arms (HA) of 30 or 40 bp: HA30trca or HA40trca (60 nM), crRNAtrca (6 µM), HiFi DNA assembly master mix (NEB, #E2621S) at 0.5X final concentration, in KGB buffer. Reactions of 5 µL were assembled and incubated for 1 h at 37 °C. Following HDR, 0.5 µL of the reaction was added to an 8 µL CFS reaction and absorbance was monitored real-time at 570 nm, using a plate reader at 37 °C.

### RePAIR

RePAIR reactions were assembled in a 5 µL final volume in KGB buffer (see above): 2 µM Cas12a, 10 mM MgCl_2_, 20 nM of truncated reporter, 60 nM of RePAIR proximal donor strand, 2 U/µL of RNAse inhibitors, 1.25 µL of ligase (final concentration of 0.5X, NEB, M0370S). If RePAIR was performed using a synthetic DNAcrRNA or using the output of an isothermal amplification, 1 U/µL of T7 RNA polymerase (Thermofischer, EP0111) and 2 mM NTPs (NEB, N0450S) were supplemented.

Finally, 100 nM of DNAcrRNA for LacZa/BFP/RFP/GFP or 57.5 nM of DNAcrRNA for Trehalase or 0.25 µL of crRNA/DNAcrRNA (at specified concentrations for sensitivity) or 5% v/v outputs of isothermal amplification were added to the RePAIR mix and incubated for 1 h at 37 °C unless specified otherwise.

RePAIR proximal donor strands (top and bottom) were ordered as ssDNA, resuspended in water at 100 µM and annealed together in a 1X PBS solution to a final concentration of 20 µM before use.

### Cell-free system

NEB PURExpress (#E6800L) was used as the only source of cell-free system (CFS) in this paper. Cell-free reactions where prepared according to the manufacturer’s protocol (40% solution A, 30% solution B), supplemented with 0.5% v/v RNase inhibitor (NEB M0314S).

#### For colorimetric output reactions

All colorimetric CFS reactions were supplemented with 0.2 µL of 25 mg/mL CPRG (chlorophenol red-b-D-galactopyranoside, Roche #10884308001) and 0.5 µL of LacZΩ in each 8 µL. LacZΩ was produced by an overnight (o/n) CFS reaction, containing 5 ng/µL of plasmid encoding the omega subunit of LacZ (LacZΩ), and supplemented directly in the CFS reactions for colorimetric outputs.

Filter papers (Whatman #Z241067) were blocked overnight in 5% BSA, then dried. A 2 mm biopsy punch (Miltex, VWR CA-95039-098) was used to cut a paper-disc from the prepared filter paper, which was then placed into reaction wells of a 384-well plate. 1.8 µL of each reaction in technical triplicate were added on paper, the plate was sealed with a clear plastic film (Sarstedt 95.1994) and incubated at 37 °C in the plate reader for absorbance monitoring at 570 nm.

#### For fluorescent output reactions

0.5 µL of the outputs reaction of RePAIR was added to an 8 µL CFS reaction set as described above. The reaction was divided into technical duplicates of 3.8 µL, loaded into the 384-well plate and incubated at 37 °C in the plate reader for fluorescence monitoring at Ex: 399/20 nm and Em: 456/20 nm for BFP, at Ex: 584/10 nm and Em: 607/10 nm for RFP, and at Ex: 485/20 nm and Em: 530/20 nm for GFP.

#### For electrochemical output reactions

0.5 µL of the outputs reaction of RePAIR was added to an 8 µL CFS reaction set as described above and supplemented with the trehalase enzyme substrate trehalose (BioShop, TRE222) at 20 mM. Reactions were incubated for 1 or 2 h at a constant temperature of 37 °C in a thermocycler. Following incubation, 0.7 µL was pipetted and measured with a glucose meter (Bayer Contour Blood Glucose Monitoring System - Model 9545 C)^28^. Each measurement was performed three times to achieve technical triplicate measurements for each reaction.

### CRISPR FnCas12a *trans*-cleavage

RT-HDA or HDA amplification were set up as described above and incubated for 90 min prior to the readout reaction. DNAseAlert (IDT #11-04-03-04), used as reporter ssDNA, was rehydrated in the 10X buffer at 3.33 µM. Reactions were set up in 13 µL final volume, containing: 100 nM of the activator dsDNA, 150 nM of the reporter ssDNA, 10 mM of MgCl_2_, 2 mM NTPs, 1 U/µL of T7 RNA polymerase, 2 U/µL of RNAse inhibitors, 2 µM FnCas12a. The product of the amplification was added at 5% v/v and reaction volume was completed to 13 µL with KGB buffer. 4 µL of each reaction was dispensed in triplicate in a 384-well plate and monitored over time using fluorescence at 536 nm / 576 nm.

### CRISPR LwCas13a *trans*-cleavage

RT-HDA or HDA amplification reactions were set up as described above and incubated for 90 min prior to the readout reaction. RNaseAlert_v2 (ThermoFisher #4479768), used as reporter ssRNA, was rehydrated in the 10X buffer at 3.33 uM. Reactions were set up in 13 µL final volume, containing: 100 nM of the activator RNA, 150 nM of the reporter RNA, 10 mM of MgCl_2_, 2 mM NTPs, 1 U/µL of T7 RNA polymerase, 2 U/µL of RNAse inhibitors, 2 µM FnCas12a. The product of the amplification was added at 5% v/v and reaction volume was completed to 13 µL with KGB buffer. 4 µL of each reaction was dispensed in triplicate in 384-well plate and monitored over time using fluorescence at 490 nm / 520 nm.

### Statistics and reproducibility

Unless otherwise indicated, experimental data sets were compared using two-tailed unpaired t tests. All statistical analysis and graphing were done using GraphPad Prism 7 software. No statistical method was used to predetermine sample size. No data was excluded from the analysis. For DWV and IAPV testing, investigators were blinded to the viral status of honey bee samples during experimental and data analysis steps.

### Cultured viruses and Bees

Cultured viruses and infected bees (2-days old, female, Apis *mellifera*) were produced as previously described^45,46^. Briefly, because no honey bee cell line currently exists for the production of large quantities of pure viral stocks^50^, virus inocula are prepared via amplification in vivo in honey bee pupae. Honey bee virus stocks were identical to those described in Hsieh et al. 2020^45,51^, which were isolated in Carrillo-Tripp et al. 2016, and Geffre et al. 2020^46,52^. Briefly, purified honey bee virus isolates were injected directly into white eyed honey bee pupae from conventionally-managed honey bee colonies at the University of Illinois Bee Research Facility. Injected pupae were incubated for 3 days in an incubator at 34 °C to allow virus propagation, after which pupae were collected and stored at −80 °C until processing. Pupae were then homogenized and virus particles precipitated and extracted with polyethylene glycol. This stock was then characterized for virus content and quantity and used in experimental infections. For this study, the cultured DWV stock was estimated to be >95% DWV with <5% IAPV contamination; the cultured IAPV stock was estimated at >99% IAPV.

Whole bees with IAPV infections were generated using methods identical to those used above. As described in these studies, this method produces bees infected with elevated IAPV titers. However, because most honey bees are hosts to background virus infections, other off-target viruses are often also detected in their tissues. Here, we placed 35 newly emerged honey bees into sterilized acrylic cages, after which they were fed a diet containing cultured virus particles mixed into 600 µl of sterile 30% sucrose solution. After this diet was consumed, the bees were fed *ad libitum* with sterile sucrose solution. Past experiments have shown that virus induced mortality occurs between approximately 48–72 h post-inoculation^46,51^. Therefore, to collect living bees with the highest probability of active infection, live bees were randomly collected from inoculated cages 36 h post-inoculation. Bees were collected, trizol inactivated, frozen on dry ice, then stored in centrifuge tubes at −80 °C until processing (CFIA application 2019-11793-1, CCM:2019:103295).

Analysis of the diagnostic application of FACTOR on bees was performed using the MedCalc online statistical tool at https://www.medcalc.org/calc/diagnostic_test.php.

### Ethical statement

Honey bee rearing and viral infection practices complied with all ethical regulations and guidelines.

### Cas12a purification

pET15-FnCas12a plasmid was transformed into BL21 cells (DE3, NEB, C2527I). 1 L of media [LB Lennox Broth, 50 µg/mL Ampicillin, 1% glucose] in a 2 L baffled flask (Tunair; SS-6001C No-Baffle Flask Kit (Dri-Gauze)) was inoculated with a starter culture and grown in a shaker (New Brunswick™; Innova® 44/44 R) at 37 °C − 200 RPM until OD600 0.6 was reached (ThermoScientific Spectrophotometer). 0.5 mM IPTG (BioShop) was then added to the culture for induction, followed by 18 h at 18 °C − 200 RPM (New Brunswick, Innova® 44/44 R). Culture was then spun down for 25 min at 4 °C − 8000 RPM using a JLA8.100 rotor (Beckman Coulter; Avanti JXN-26) and the supernatant was decanted. The pellet was resuspended in 30 mL filter-sterilized Lysis Buffer, pH 7.5 [0.005 M imidazole, 0.5 M NaCl, 0.05 M HEPES, 5% (v/v) glycerol, 0.5 mM TCEP, 0.1 mM PMSF], homogenized by vortexing, sonicated (Qsonica Sonicator) on ice using a 6.4 mm probe for 6 min (1 s ON, 1 s OFF, 50% amplitude), and spun down using a JA-14.50 rotor (Beckman Coulter; Avanti JXN-26) for 45 min at 4 °C – 17000 RCF. The supernatant was then passed through a 0.45 µm filter (MilliporeSigma Syringe Filters), followed by a 0.22 µm filter and kept at 4 °C. The lysate was then purified using the AKTA Pure Chromatography System (GE Healthcare, FPLC) on a 1 mL HisTrap FF Column. The column was first equilibrated with 5 columnar volumes (CVs) of filter-sterilized Binding Buffer, pH 8.2 [0.015 M imidazole, 0.5 M NaCl, 0.02 M HEPES, 5%(v/v) glycerol]. Lysate was loaded onto the column, which was then washed using 20 CVs of Binding Buffer, and eluted using 10-20 CVs of filter-sterilized Elution Buffer, pH 8.2 [0.5 M imidazole, 0.5 M NaCl, 0.02 M HEPES, 5% (v/v) glycerol]. The elution fractions corresponding to the chromatography peak were tested using Coomassie Brilliant Blue staining of 10% SDS-PAGE gels. Fractions showing expression were consolidated in an Amicon Ultra-15 Centrifugal Filter Unit 50 kDa (MilliporeSigma) and washed four times in Storage Buffer, pH 7.5 [0.5 M NaCl, 0.02 M HEPES, 5% (v/v) glycerol]. The purified protein was aliquoted and flash frozen in liquid nitrogen.

### RT-qPCR

To estimate reaction efficiency, DNA templates were in vitro transcribed with the HiScribe T7 Quick High Yield RNA synthesis kit (NEB, E2040S) according to the manufacturer’s instruction. 100 ng of RNA was used as template for cDNA synthesis using the Superscript IV VILO kit (ThermoFisher #11756050) and 10x serial dilutions were performed and ran alongside samples being tested to establish standard curves. The equation of the slope for each standard curve was used to establish the correlation between Ct values and viral copy number per µL. A Ct of 38 was used as the standard cut-off for all positive reactions.

Extracted RNAs (whole bees or viral aliquots) were quantified using a nanodrop (ThermoFisher, #ND-ONE-W), diluted in RNase-free water and 100 ng RNA input was used for cDNA synthesis. For RT-qPCR setup, PowerUP SYBR green master mix (ThermoFisher #25742) and appropriate primer sets (Supplementary Data 1) were prepared for 10 µL total reactions in a 384-well plate format, with 2 µL of cDNA input, or controls, added to each well. Real-time PCR was performed on a Bio-Rad CFX-384 system with standard cycling and melt curve analysis. Ct of 38 was used as the standard cutoff.

### Reporting summary

Further information on research design is available in the Nature Portfolio Reporting Summary linked to this article.

## Supplementary information

Supplementary Information

Description of Additional Supplementary Files

Supplementary Data 1

Reporting Summary

## Data Availability

Source data are provided with this paper.

## Source data

Source Data
