# Supplementary material for: CRISPR-induced DNA reorganization for multiplexed nucleic acid detection
Source: Nat Commun. 2023 Mar 17;14:1505. doi: 10.1038/s41467-023-36874-6 (PMC10022571; doi:10.1038/s41467-023-36874-6)
Supplement: Supplementary file 1 — Supplementary Information [file 41467_2023_36874_MOESM1_ESM.pdf]

Supplementary Information for:

CRISPR-induced DNA reorganization for multiplexed nucleic acid detection

Margot Karlikow[1*], Evan Amalfitano[1], Xiaolong Yang[1], Jennifer Doucet[1], Abigail Chapman[2], Peivand Sadat Moussavi[1], Paige Homme[1], Polina Sutyrina[1], Winston Chan[1], Sofia Lemak[3], Alexander F. Yakunin[3,4], Adam G. Dolezal[5], Shana Kelley[1,6,7], Leonard J. Foster[2], Brock Harpur[8], Keith Pardee[1,9*]

[1] Department of Pharmaceutical Sciences, Leslie Dan Faculty of Pharmacy, University of Toronto, Toronto, ON, Canada M5S3M2

[2] Department of Biochemistry & Molecular Biology, Michael Smith Laboratories, University of British Columbia, Vancouver, BC, Canada

[3] Department of Chemical Engineering and Applied Chemistry, University of Toronto, Toronto, Ontario, M5S 3E5, Canada

[4] Centre for Environmental Biotechnology, School of Natural Sciences, Bangor University, Bangor, Gwynedd LL57 2UW, UK

[5] Department of Entomology, University of Illinois at Urbana–Champaign, Urbana, IL 61801

[6] Institute of Biomedical Engineering, University of Toronto, Toronto, Canada M5S 3G9.

[7] Department of Chemistry, Faculty of Arts and Science, University of Toronto, Toronto, Canada M5S 3H4.

[8] Department of Entomology, Purdue University, 901 W State Street, West Lafayette, IN 47907, USA.

[9] Department of Mechanical and Industrial Engineering, University of Toronto, Toronto, ON, M5S 1A1, Canada.

[*] Co-corresponding authors. Email: margot.karlikow@gmail.com (M.K.) or keith.pardee@utoronto.ca (K.P.)

Table of Contents

1 Supplementary Figures:

Supplementary Fig. 1: Functionalized amplification product of FACT

Supplementary Fig. 2: Homologous direct recombination (HDR) based CRISPR induced gene-circuit output

Supplementary Fig. 3: Validation of RePAIR system

Supplementary Fig. 4: RePAIR time-course

Supplementary Fig. 5: Sensitivity of FACTOR

Supplementary Fig. 6: RT-qPCR results for compared diagnostic application

Supplementary Fig. 7: Potential use of FACTOR for molecular labelling.

2 Supplementary Data 1:

Sequences

1) Supplementary figures:

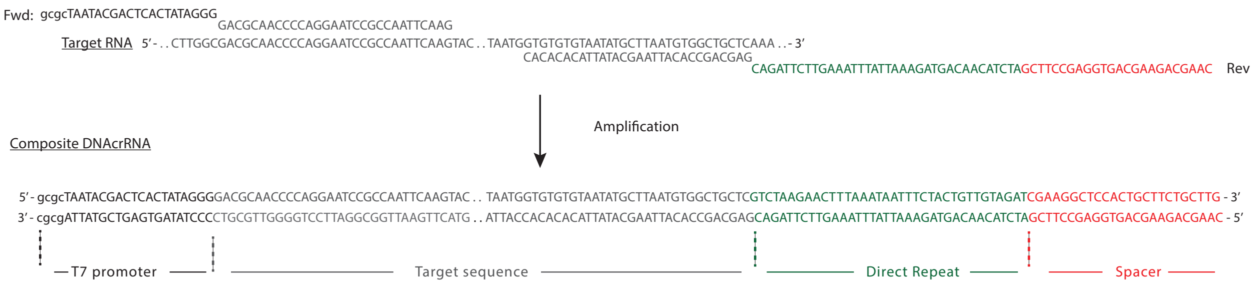

Supplementary Fig. 1: Sequence of the functionalized amplification product of FACT. Using programmable primers (Fwd and Rev) targeting a specific PAM-free nucleic acid of interest, HDA-based isothermal amplification generates a composite DNAcrRNA. The functionalized amplicon now contains a T7 RNA polymerase promoter (black), the PAM-free target sequence (grey), a direct repeat (green) linked to the CRISPR enzyme of interest and a spacer sequence (red), enabling targeted sequence specific *cis*-cleavage readout events.

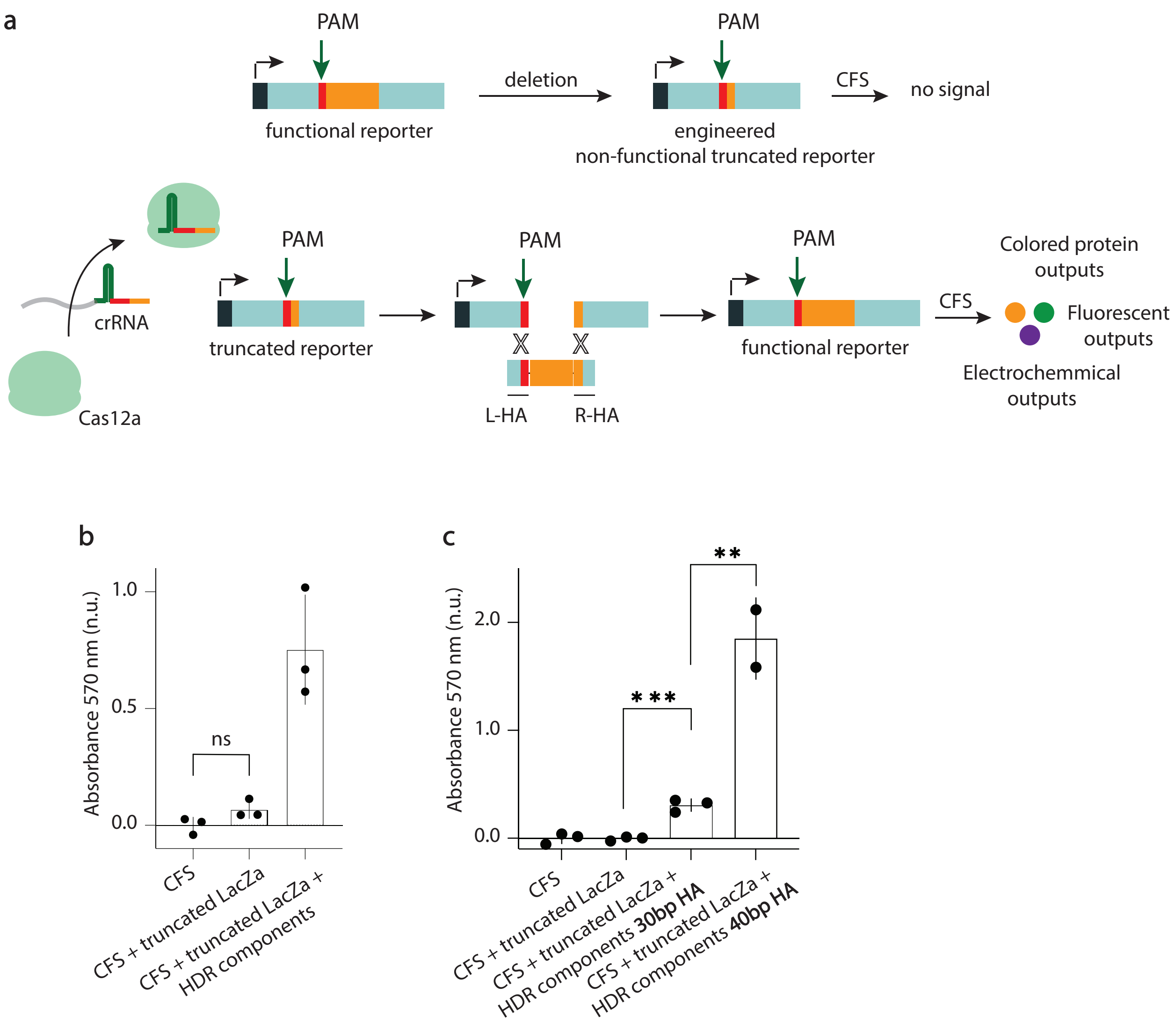

Supplementary Fig. 2: Homology directed repair (HDR)-based CRISPR induced gene circuit output. a. A schematic of the molecular components and process: a non-functional reporter was engineered by truncating the coding sequence of a reporter enzyme, here LacZα, impairing the generation of a functional readout in CFS. In presence of (*i*) Cas12a, (*ii*) a crRNA programmed to target the non-functional reporter and (*iii*) a dsDNA donor containing the missing nucleotides as well as homologous arms: left (L-HA) and right (R-HA), a functional reporter is assembled that can generate a readout of interest in CFS. **b.** Using LacZα as a reporter, HDR was performed followed by addition of the product to CFS. Absorbance of the reaction was monitored (570 nm) over time as a readout of the successful recombination of LacZα. **c.** Comparison of HDR using donor dsDNA with HA of 30 bp or 40 bp and absorbance at 570 nm was monitored over time as a readout of the successful recombination of LacZα. **b.** and **c.** Representative experiment of biological triplicates (*n*=3) displaying the final time point at 240 min in the CFS. Error bars: mean ± SD. Statistical test used for data analysis was a two-tailed unpaired t tests. ns: not significant. **: p=0.0047. ***: p=0.0009.

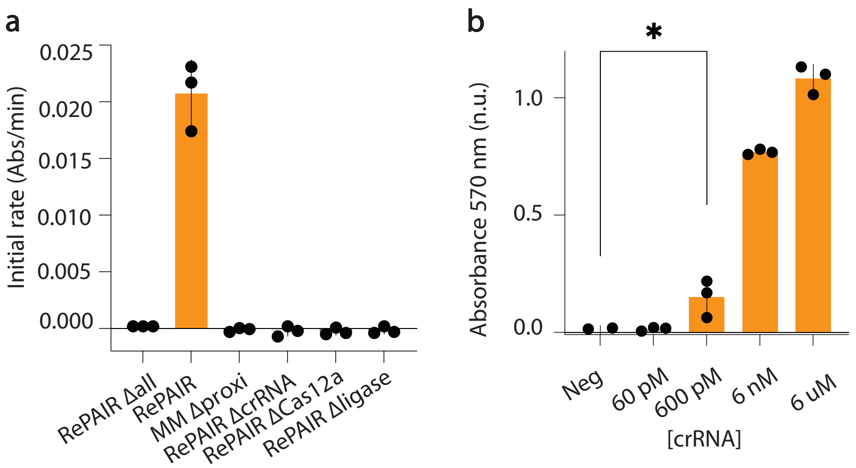

Supplementary Fig. 3: Validation of the RePAIR system. a. Initial rate within 1 hour of CFS of the absorbance at 570 nm initiated by the DNA reorganization of LacZα (induced by a crRNAtrc3α). All the components of RePAIR were sequentially removed. **b.** Limit of detection of the crRNA inducing RePAIR of LacZα, within the first 2 hours of CFS. **a.** and **b.** Representative experiments of biological triplicates (*n*=3). Statistical test used for data analysis was a two-tailed unpaired t tests. *: p=0.0375. Error bars: mean ± SD.

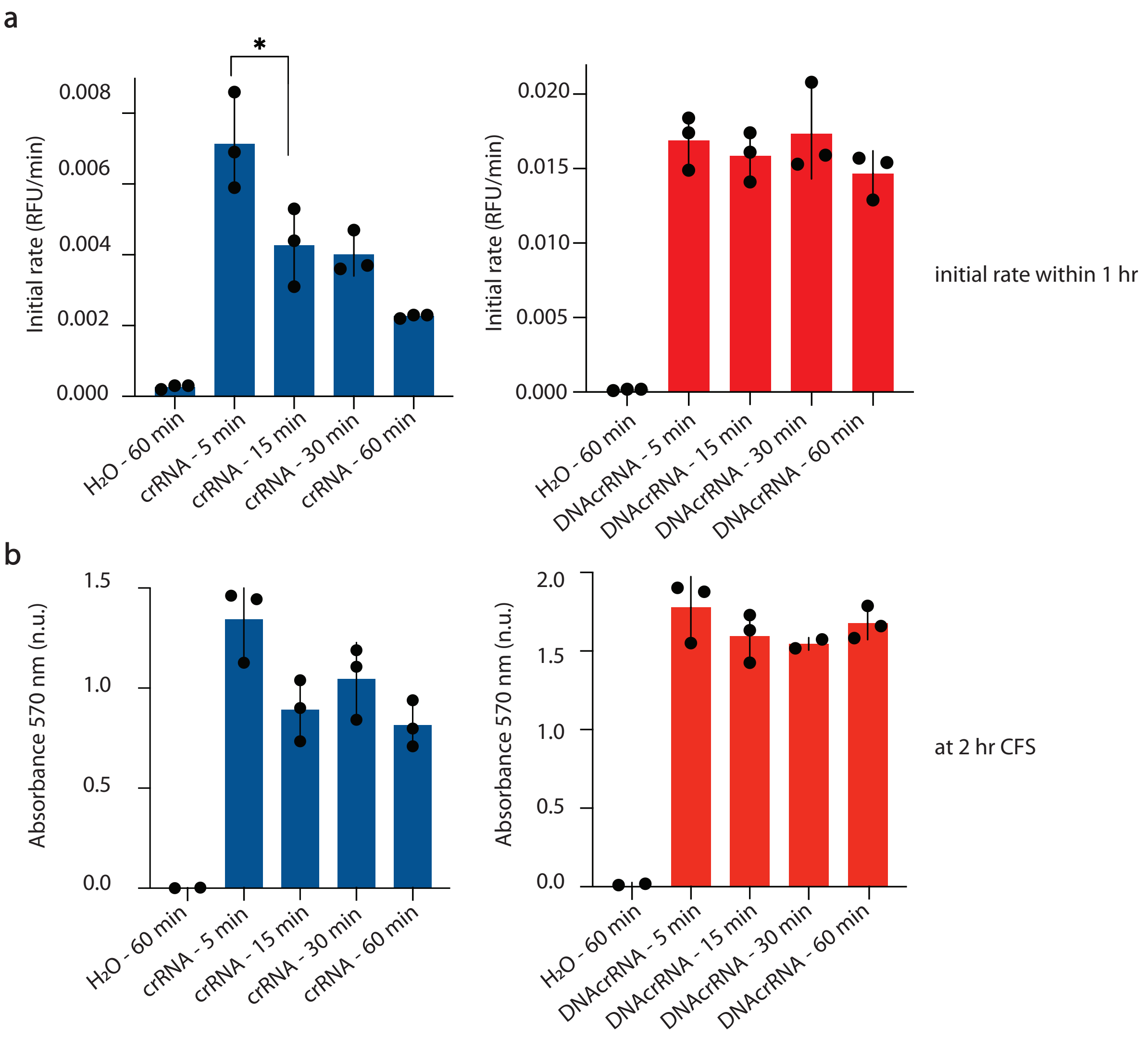

Supplementary Fig. 4: RePAIR time-course. a. Maximal initial rate within 1 hour of CFS of absorbance increase at 570 nm, using LacZα RePAIR reactions incubated for 5-, 15-, 30-, or 60 min at 37°C. RePAIR reactions were initiated using crRNAtrc3α (blue, left) or DNAcrRNAtrc3α (red, right). **b.** At 2 hours of CFS, absorbance at 570 nm is shown, displaying levels of functional LacZ in the CFS. a. and b. are representative experiments of biological triplicates (*n*=3). b. normalized units (n.u.). Error bars: mean ± SD. Statistical test used for data analysis was a two-tailed unpaired t tests. *: p=0.0475.

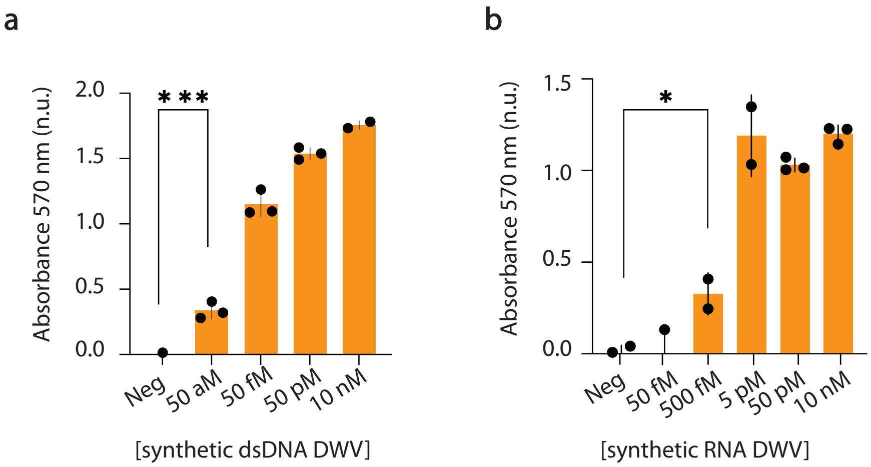

Supplementary Fig. 5: Determination of sensitivity for FACTOR. a. A stock of synthetic DNA encoding for DWV was used at the specified concentrations for FACTOR (isothermal amplification, and RePAIR). **b.** A stock of synthetic RNA encoding for DWV was used at the specified concentrations for FACTOR. Error bars: mean ± SD. Experiments were run in biological triplicate (*n*=3), each containing a technical triplicate. Data are representative experiments of an independent technical triplicate. Statistical test used for data analysis was a two-tailed unpaired t tests. ***: p=0.0009. *: p=0.0185.

a

DWV cultured viruses	Av. Ct value IAPV	Av. Ct value DWV
	26.16	34.48
corresponding viral copy / uL	1.42×10^6	4.33×10^5

IAPV cultured viruses	Av. Ct value IAPV	Av. Ct value DWV
	15.05	N.D.
corresponding viral copy / uL	1.79×10^9	N.D.

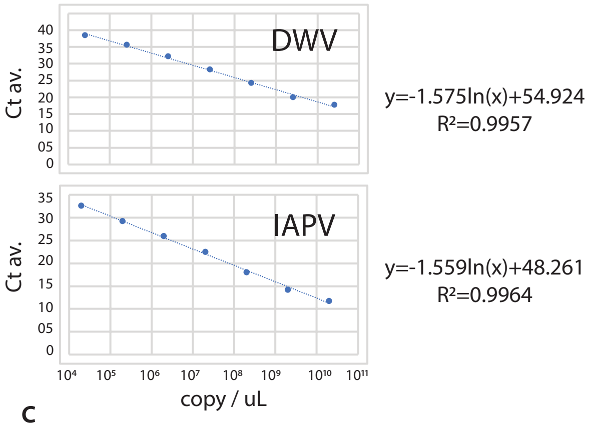

b

Whole bees	Av. Ct value IAPV	Av. Ct value DWV
Bee 1	31.37	N.D.
Bee 2	33.42	N.D.
Bee 3	31.32	N.D.
Bee 4	30.31	N.D.
Bee 5	34.11	N.D.
Bee 6	18.03	N.D.
Bee 7	17.56	N.D.
Bee 8	18.80	N.D.
Bee 9	18.04	N.D.
Bee 10	18.68	N.D.
Bee 11	N.D.	32.49
Bee 12	N.D.	N.D.
Bee 13	N.D.	33.54
Bee 14	25.59	34.39
Bee 15	> 38	> 38
Bee 16	24.79	26.53
Bee 17	> 38	24.88
Bee 18	N.D.	N.D.
Bee 19	N.D.	N.D.

c

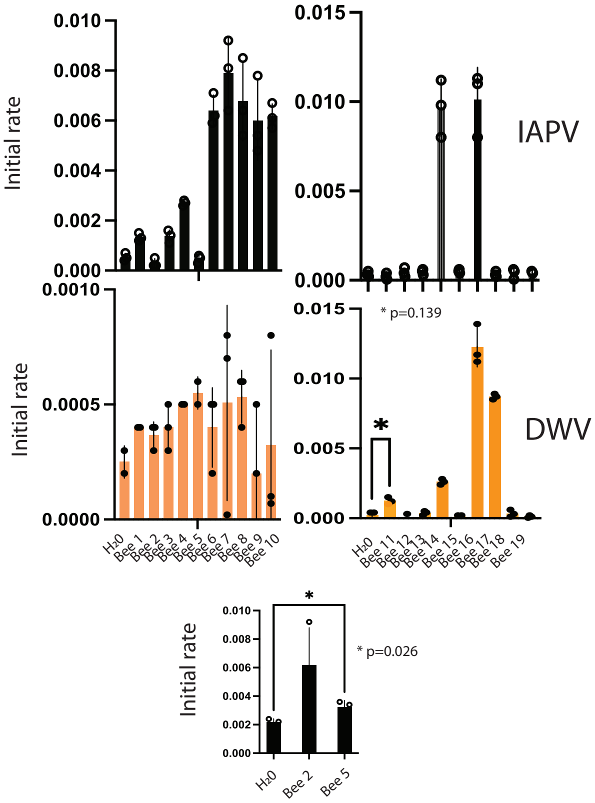

Supplementary Fig. 6: RT-qPCR results for comparison of FACTOR with the gold standard diagnostic. a. RT-qPCR was performed on extracted RNA from cultured viruses. **b.** RT-qPCR was performed on extracted RNA from whole bees. Corresponding Ct values were calculated based on standard curves. Discrepancies are indicated in red. **c.** Initial rates of the FACTOR diagnostic reactions on the 19 bees used in the comparison to the RT-qPCR data presented in b. Data displayed in c. are the 19 independent RePAIR experiments (*n*=1, Bee 1 to 19), each containing a technical triplicate. Error bars: SD. Statistical test used for data analysis was a two-tailed unpaired t tests.

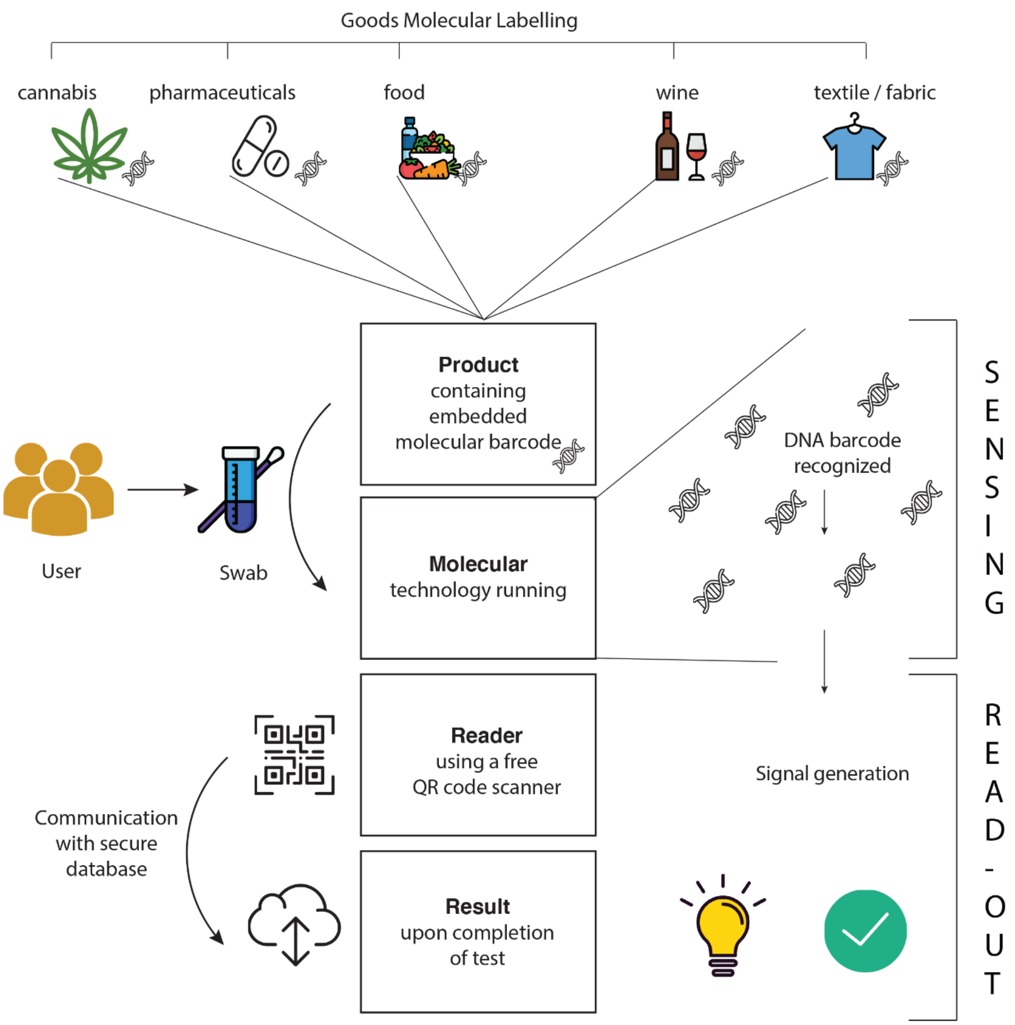

Supplementary Fig. 7: Potential use of FACTOR for molecular labelling. DNAcrRNA can be spread or embedded on/into various products. When needed, the user can collect the DNAcrRNA through a gentle swab of the product for authentication with a FACTOR reaction.

2) Supplementary Data 1: Sequences

Sequences can be found in the excel file provided with the manuscript as an independent supplementary information, for ease of use.
