# Supplementary material for: CRISPR-induced DNA reorganization for multiplexed nucleic acid detection
Source: Nat Commun. 2023 Mar 17;14:1505. doi: 10.1038/s41467-023-36874-6 (PMC10022571; doi:10.1038/s41467-023-36874-6)
Supplement: Supplementary file 2 — Description of Additional Supplementary Files [file 41467_2023_36874_MOESM2_ESM.pdf]

Description of Additional Supplementary Files

Title: Supplementary Data 1

Description: Information on all sequences used in this study.
