# Supplementary material for: CRISPR-induced DNA reorganization for multiplexed nucleic acid detection
Source: Nat Commun. 2023 Mar 17;14:1505. doi: 10.1038/s41467-023-36874-6 (PMC10022571; doi:10.1038/s41467-023-36874-6)
Supplement: Supplementary file 4 — Reporting Summary [file 41467_2023_36874_MOESM4_ESM.pdf]

nature portfolio

Corresponding author(s): Karlikow M, Pardee K

Last updated by author(s): Apr 5, 2022

Reporting Summary

Nature Portfolio wishes to improve the reproducibility of the work that we publish. This form provides structure for consistency and transparency in reporting. For further information on Nature Portfolio policies, see our Editorial Policies and the Editorial Policy Checklist.

Statistics

For all statistical analyses, confirm that the following items are present in the figure legend, table legend, main text, or Methods section.

n/a	Confirmed	
☐	☒	The exact sample size (n) for each experimental group/condition, given as a discrete number and unit of measurement
☐	☒	A statement on whether measurements were taken from distinct samples or whether the same sample was measured repeatedly
☐	☒	The statistical test(s) used AND whether they are one- or two-sided<br>*Only common tests should be described solely by name; describe more complex techniques in the Methods section.*
☒	☐	A description of all covariates tested
☒	☐	A description of any assumptions or corrections, such as tests of normality and adjustment for multiple comparisons
☐	☒	A full description of the statistical parameters including central tendency (e.g. means) or other basic estimates (e.g. regression coefficient) AND variation (e.g. standard deviation) or associated estimates of uncertainty (e.g. confidence intervals)
☐	☒	For null hypothesis testing, the test statistic (e.g. F, t, r) with confidence intervals, effect sizes, degrees of freedom and P value noted<br>*Give P values as exact values whenever suitable.*
☒	☐	For Bayesian analysis, information on the choice of priors and Markov chain Monte Carlo settings
☒	☐	For hierarchical and complex designs, identification of the appropriate level for tests and full reporting of outcomes
☒	☐	Estimates of effect sizes (e.g. Cohen's d, Pearson's r), indicating how they were calculated

Our web collection on statistics for biologists contains articles on many of the points above.

Software and code

Policy information about availability of computer code

Data collection	Gen5 3.02 (BioTek Instruments Inc.) was used to collect data from plate reader assays.
Data analysis	Prism 7 (GraphPad Software) was used to perform statistical analysis. Medcalc was used for the statistical analysis of the diagnostic performances.

For manuscripts utilizing custom algorithms or software that are central to the research but not yet described in published literature, software must be made available to editors and reviewers. We strongly encourage code deposition in a community repository (e.g. GitHub). See the Nature Portfolio guidelines for submitting code & software for further information.

Data

Policy information about availability of data

All manuscripts must include a data availability statement. This statement should provide the following information, where applicable:

- Accession codes, unique identifiers, or web links for publicly available datasets
- A description of any restrictions on data availability
- For clinical datasets or third party data, please ensure that the statement adheres to our policy

Supplementary data is available with the manuscript. All data generated or analyzed during this work will be made available, upon request. Source data are provided with this paper.

Field-specific reporting

Please select the one below that is the best fit for your research. If you are not sure, read the appropriate sections before making your selection.

- [x] Life sciences
- [ ] Behavioural & social sciences
- [ ] Ecological, evolutionary & environmental sciences

For a reference copy of the document with all sections, see nature.com/documents/nr-reporting-summary-flat.pdf

Life sciences study design

All studies must disclose on these points even when the disclosure is negative.

Sample size	Samples were chosen according to replicate convention (n=3), as detailed below (Replication).
Data exclusions	No data was excluded from the analyses.
Replication	All in vitro studies were independently performed in triplicates. Trial on extracted RNA of bees samples was performed as one experiment on 19 independent bees, comparing during the same day our diagnostic technology to the gold standard RT-qPCR.
Randomization	This paper describes the development of a new technology, therefore a large randomized trial was not included.
Blinding	No sample allocation to groups was performed, bees were received already infected or not, and their infectious status was considered as unknown, so blinding was not relevant to this study.

Reporting for specific materials, systems and methods

We require information from authors about some types of materials, experimental systems and methods used in many studies. Here, indicate whether each material, system or method listed is relevant to your study. If you are not sure if a list item applies to your research, read the appropriate section before selecting a response.

Materials & experimental systems

n/a	Involved in the study	
x		Antibodies
x		Eukaryotic cell lines
x		Palaeontology and archaeology
	x	Animals and other organisms
x		Human research participants
x		Clinical data
x		Dual use research of concern

Methods

n/a	Involved in the study	
x		ChIP-seq
x		Flow cytometry
x		MRI-based neuroimaging

Animals and other organisms

Policy information about studies involving animals; ARRIVE guidelines recommended for reporting animal research

Laboratory animals	The study involved laboratory animal 2-days old female honey bees Apis mellifera.
Wild animals	No wild animal were used in this study.
Field-collected samples	No field-collected samples were used in this study.
Ethics oversight	no ethical approval or guidance was required.

Note that full information on the approval of the study protocol must also be provided in the manuscript.
